# Preceptor engagement in distributed medical school campuses

**Published:** 2015-12-11

**Authors:** Thomas Piggott, Cathy Morris, Michael Lee-Poy

**Affiliations:** 1 Michael G DeGroote School of Medicine, Waterloo Regional Campus, McMaster University; 2 Department of Clinical Epidemiology and Biostatistics, McMaster University

## Abstract

**Background:**

There is increasing interest in distributed medical campuses and engagement of physicians in these communities. To date, there has been suboptimal recruitment of physicians to participate in medical education at distributed campuses. The purpose of this project was to identify barriers to engagement in medical education by community physicians in the geographical catchment of the Waterloo Regional Campus of McMaster.

**Method:**

In-depth, semi-structured, qualitative interviews were conducted with physicians not involved in teaching. Interview recordings were transcribed and analyzed using a closed-loop, iterative coding methodology and thematic analysis was performed. Interviews were conducted until thematic saturation was achieved.

**Results:**

Six interviews were conducted and coded. Nine key themes emerged: academic centre versus distributed sites, interest in teaching, financial considerations, administrative barriers, medical experience and knowledge currency, practice environment and schedule, training on teaching, setting up systems for learners in distributed campus settings, and student engagement and medical learner level.

**Conclusions:**

Barriers to engagement in teaching primarily focused on differences in job structure in the community, administrative barriers both at the hospital and through the medical school, and lack of knowledge on how to teach. As medical schools look to expand the capacity of distributed campuses, misperceptions should be addressed and opportunities to improve engagement should be further explored.

## Introduction

With increasing student enrollment, medical schools are turning to distributed campuses for expansion.^1^ Distributed campuses are medical school training sites located at a distance from the primary academic centre, while still retaining linked curriculum. There are now numerous examples of distributed campuses, including for example: the Northern Medical Program of University of British Columbia, Dalhousie Medicine New Brunswick in Saint John, and the Waterloo Regional Campus of McMaster University.^2^,^3^,^4^ Recruitment of physicians to serve as preceptors at distributed campuses has had mixed results. A subset of physicians has reportedly been quick to engage, while others have been reluctant, leaving a need for preceptorship.

A review of the literature revealed little published in the realm of barriers to preceptor engagement, and less with respect to those barriers faced in distributed campus settings. In a qualitative study conducted in England, barriers to teaching and learning identified by physicians included change resistance and an uncertainty of their potential roles as educators.^5^ A research project carried out by the Northern Medical Program of UBC identified that the distributed campus created new relationships amongst the physician community, improving community cohesion and social capital.^6^ Finally, a study in Australia reported that rewards reported by general practitioners involved in teaching included an enhancement to teamwork and morale within the practice; additionally they report negative aspects of teaching including reduced productivity and increased time efforts.^7^

We have noticed several potential barriers: financial loss involved with having medical clerks in practice; lack of awareness about opportunities to preceptor with the campus; hesitancy to be exposed to the questioning of medical decisions that medical students bring; disenchantment at previous teaching experiences; having left an academic centre to escape burdens of teaching in a non-medical school city; and necessity to maintain a grasp on the most current medical literature. However, the precise factors and barriers affecting engagement in a distributed medical school campus have not been systematically explored.

## Methods

McMaster University Health Sciences Research Ethics Board approval was obtained for a study involving human participants. Semi-structured, qualitative, open-ended interviews were conducted with a representative sample of physicians in the communities serviced by the Waterloo Regional Campus of McMaster University. The sample was determined by identifying physicians from the College of Physicians and Surgeons of Ontario Doctor Search who were practicing in 5 communities within the campus region and in cross-referencing to faculty appointments, choosing those who were not presently engaged in clinical or academic teaching. Individuals from this list of physicians who had previously expressed interest in teaching but were not yet engaged in medical education were excluded as potential respondents. Stratification of the group of potential respondents by specialty and years of practice was performed.

Potential interviewees were contacted at random from the stratified groups and invited to participate in a semi-structured 45–60 minute interview. Out of 31 physicians that were invited to participate, nine expressed interest in participating and ultimately six interviews were conducted. Three individuals that initially expressed an interest in participating were not interviewed due to an inability to schedule.

Interviews were conducted using an in-depth, semi-structured, qualitative interviewing method. All interviews were conducted by the primary investigator (Piggott). Six pre-determined questions formed the framework for the interviews (Appendix A). Interviews focused on identifying experiences with medical education, barriers to engagement, and potential actions that distributed campuses may take to increase engagement. Interviews were recorded and transcribed for analysis. Analysis was conducted in an iterative manner after each interview. Interview transcriptions were de-identified and uploaded into Dedoose, an online qualitative data analysis software.^8^ Thematic analysis of the interview transcripts was performed in Dedoose using a closed-loop, iterative coding methodology.^9^ Quotes identified from thematic analysis were assessed for relevance and quotes and themes were reviewed and analyzed by all authors on this paper.

## Results

Six physicians from varied disciplines and not actively engaged in teaching were interviewed. The average length of practice was 10.7 years, with the minimum being 1.5 years and the maximum 26 years. Closed-loop, iterative coding of the interview transcripts led to the creation of 34 codes. The codes identified are listed in Table 1. Thematic analysis was performed using the key quotes identified and coded with the 34 codes generated. Nine key themes were identified with a total of 97 key quotations extracted from the transcripts. Description of each of the themes is provided below.

### Academic centre vs. distributed sites

The perceived differences between motivations to teach were identified.

The motivation for teaching in [academic centres] is not necessarily the best… I don’t want to be up all night. So what can I do? … Get into medical education…

The flip side is, the guys in academic centres, you’re not going to be there any more if you don’t teach. That will never happen here.

Additionally, respondents reflected the differences in opportunities available for medical learners at distributed sites, particularly in procedural specialties of medicine.

Whereas in community rotations generally, you get less scutt, but you get less opportunity as well.

This was attributed in part due to hospital staff and administrators at distributed campus affiliated hospitals who may not be as accommodating to the learning process.

When I first came here and you let the clerk close the incision, people would be pissed off.

### Interest in teaching

Most respondents stated that they were at least somewhat interested in teaching and believed that they would enjoy it, but that other barriers had prevented them from becoming engaged in teaching.

Sure I would rather spend an hour teaching somebody about something rather than doing a discharge summary, but you do not want to have to go teach somebody and do the discharge summary.

[Physicians who teach] are doing it sort of out of their professional duty to teach people and to help make the next generation of doctors better, but yeah you know, there is a lot of altruism, it really amazes me.

Some also acknowledged the benefits of teaching.

[One teaching experience I had a while ago] was good; I liked it; it was interesting. Interacting with people is good, and it keeps you up-to-date and makes sure you are learning and really know your knowledge.

### Financial considerations

Sentiments reflected in similar ways by most respondents were: “the amount of money that you would have to pay doctors to make it worthwhile to do it, you couldn’t afford to” and “nobody does it for money. You teach because you want to teach.” Another issue brought up by multiple respondents was that the perceived non-monetary benefits of teaching in academic centres do not necessarily exist in distributed sites.

You might say I am thinking a little bit selfish, but then go ask the university professor who doesn’t pay his secretary or office rent or doesn’t really do call and who is going to go home at 5:30 regardless, ask them to ‘pay’ 300 bucks a day [lost income] to do teaching and on top of that ask them to stay an extra hour to do all that.

### Administrative barriers

Perceived medical school and hospital administrative barriers were identified. In terms of becoming affiliated as an adjunct professor to be able to teach, one physician said “I think just making it easier, so not putting all of the hoops you have to jump through like the paperwork to become an adjunct professor.” Another respondent described unrealistic expectations by hospital administration.

I think that [the hospital administration] are supportive of us teaching, but they also want the wait [times] to be as short as possible... they want perfect.

Several respondents described that they would potentially be interested in teaching, but had not been contacted with information or an invitation to teach.

I haven’t gotten contacted by them [the medical school] either. Maybe they would like it if I did [teach], but they didn’t contact me.

### Medical experience and knowledge currency

Issues were identified around challenges for new physicians teaching.

I definitely think I am more comfortable now with a learner than when I first started. I think putting a learner with a brand new staff member is just stupid. They’ve just started and they’re battling huge adversity and adjustments in their new career and I think it’s just a bad decision to ever put a learner with a staff member in their first year.

The longer that I am in practice, and the better I am, the more comfortable I would be with teaching.

Additionally, respondents described that as more time stands between them finishing school and starting to teach, their knowledge decreases making teaching difficult and a potentially stressful experience.

So all of that medical knowledge… I mean I could get it back, but why would I? So it’s hard to impart that knowledge to people when it is not on the tip of your fingertips.

Some people do it [start teaching after many years in practice], but I could see as I get more and more into my schedule not wanting to change.

Furthermore, several respondents have reflected that they would want the latest medical evidence and that teaching any less is a disservice to the medical learners.

You can [bs] anybody. When [a medical learner] is asking you a question, you can convince people that pretty much anything is true. But that is not the goal of medical education; I think that the goal is to give people an evidenced-based framework. Giving people a plausible explanation that actually is not correct is not helping someone, it’s actually harming them.

### Practice environment and schedule

A common statement by respondents was that the capacity to teach is very dependent on the medical practice environment and other stressors of patient care.

Surviving the shift and taking care of patients safely was definitely first and foremost and if the learner can get anything from it, it’s a bonus.

Additionally, respondents reflected varied opinions on the impacts that having learners would have on their schedule.

If I was starting to run behind and had a student here, who didn’t really get what was going on, that we were running behind, and was asking me questions. I would just be like ‘Stop! Go away!’

I’m not sure if I would do a good job [teaching]. I am not sure if I could manage the workflow. I mean I have never done it before, anything that you’ve never done before you feel a bit more nervous and apprehensive about.

I think like anything, teaching is something you have to get experience and get good at. At first you have to book less people, but then you probably could book regularly.

### Training on teaching

Respondents felt that they had spent significant time in residency engaged in clinical teaching.

When you’re a resident, all you do is teach. Teach, teach, teach. And that’s not that bad. If somebody asks what you are doing, you just sit down and talk about it.

You are a pretty bad fourth-year resident, if you’re not talking to anybody [medical students] and doing teaching. It is part of the training expectation.

Despite several comments that there is significant teaching in residency, several respondents reflected that they felt ill equipped to teach.

The truth is as a staff member I have never been formally taught how to teach residents. I’ve never taken a course in how to teach residents, how to teach medical students. I only have training as a doctor. I am not an educator.

Solutions suggested included providing education around teaching during training, and professional development regarding teaching to practicing physicians.

Maybe there could be more teaching about teaching in residency and medical school.

Several respondents stated that they would be interested in opportunities to learn how to make teaching experience worthwhile and become good teachers.

I think that if medical schools want more teachers, then they have to take the initiative to get the professional development. Get those courses into their cities, and encourage and bring doctors in to do that.

At the same time, it was reflected that these professional development opportunities to develop teaching skills would be less desirable if they took away personal time or were costly.

I think that the cost as well as a barrier, even though no doubt doctors are wealthy, and I’m definitely not begrudging that, but to pay $1000 to go to a course on the weekend out of your own pocket, you know, that is a barrier.

I think that as a staff member, development would be really beneficial. But there are barriers to that as well, these courses are weekend courses. We have families, and I work every other weekend anyway. I am not really excited to go to a weekend course when I only have two weekends a month spent with my wife and family. So that is a barrier.

### Setting up systems for learners in distributed campus settings

Respondents stated that there are unique challenges to the distributed campus setting.

[In community rotations] it is really hard to establish a team.

Matching students with doctors based on their desire to teach, and also their experience is important.

Furthermore, respondents stated that it is important that well thought through roles, responsibilities and learning objectives accompany students on their rotations.

I don’t think he understood what responsibilities they [medical learners] should have. I remember my Dad [a rural family doctor] had a first-year resident, and he just had her following him around. And I said Dad, let her go be autonomous, let her go into the room and do the full thing. And then let her present it to you. And he said ‘oh that’s a great idea, I hadn’t thought about that.’

[The student should come with a] one page summary… so you sort of know what to focus on… What do you want to work on, what do you want to see? So if there was a shortlist with expectations from the curriculum.

### Student engagement and medical learner level

Several respondents described the importance of student interest and engagement, and of clear learning objectives if they were to be engaged in teaching.

I will tell you, the most gratifying, by far by far by far, the most gratifying educational experience that I have ever had, where I really thought that they did something useful was a nurse practitioner. Because she knew what she wanted to know and she was already quite knowledgeable.

One respondent described an experience early in her/his career, which evidently dissuaded her/him from teaching significantly later in life.

This guy [medical student previously working with me] just didn’t seem interested. I just wondered why did you decide to come, I mean it is not a mandatory rotation; nobody forced you to be here.

Another respondent described that they are less interested in teaching when the students are not enthusiastic about learning.

You have no idea how lucky you are to see this. But they’re like ‘oh what is he talking about? I have no idea.’

Remember an N=1 study is disastrous, but one of the students [that worked with me] missed several times that we had set aside. He just didn’t show. He didn’t call or anything… so the experience was very disconcerting.

Furthermore, most respondents stated that it is substantially more work for them to teach lower level learners, such as medical students compared to residents.

It depends on the level of the student. If it’s a medical student it might take more time and run your clinic overtime. Versus an R2, they might actually speed things up.

## Conclusion

Medical schools must work to increase engagement of preceptors at distributed campuses as they continue to offer more opportunities to their learners and expand in size. The preliminary results of this study have identified factors impacting the engagement of physicians to teach at a distributed campus setting including differences in job structures and practice settings in distributed sites versus academic centres, administrative barriers to teaching at both the hospital and medical school campus level, time since last being involved in teaching and dated medical knowledge. These barriers stand in contrast to the prior belief that financial barriers to teaching are the central challenge; the respondents in this study discounted this as a primary barrier to engagement in teaching.

Additionally, this study has identified potential avenues to facilitate teaching processes and increase preceptor engagement, such as having learners arrive with clear learning objectives, opportunities for professional development of teaching skills, and working with hospitals and medical schools to create structures and systems that facilitate teaching and the roles of medical learners.

This study can draw attention to the significant divide between the perception and reality of teaching at distributed medical school campuses. An in-depth discussion of this issue is outside of the scope of this study. However, several misperceptions were identified from the regional campus context. The first pertains to the availability of faculty development opportunities related to teaching. For several years now, great effort has been made to offer opportunities for faculty development within the communities of the regional campus to better facilitate access to these workshops. They have been formed and reformed based on feedback and widely advertised. However, there has generally been low attendance.

Additional misperceptions exist between physicians at academic centres and those in the community. The beliefs expressed by respondents in this study about the benefits afforded to academic physicians were largely inaccurate. Although not the purpose of this study, we have observed that there are also a number of negative misperceptions by academic physicians about the interest in teaching and motivations of community physicians.

A final misperception worth mentioning surrounds the administrative barrier reported by respondents to applying for adjunct faculty appointment. Much effort has been made to mitigate the time required to complete the appointment application: the form is less than one page and includes details regarding demographics and two questions related to interest in involvement in teaching at the undergraduate and postgraduate levels.

The conclusion raised from these three examples is that there are still significant misperceptions by community physicians surrounding engagement in medical education that may serve as a barrier to teaching. Distributed medical school campuses should make a concerted effort to identify misperceptions and work to dispel or address them in their own communities.

The present study has looked into the factors impacting engagement amongst physicians not involved in medical education. While this group represents a large and stable proportion of community physicians, we must also acknowledge that there has been an equally strong group of community physicians who have been passionate early adopters of distributed medical education. Future research into the qualities, traits, and beliefs of these community preceptors would significantly benefit the field of medical education.

As medical schools increasingly turn to distributed campus settings to address growing enrolment and offer additional opportunities to their students, we hope the information presented in this study will assist the planning efforts to increase preceptor engagement and improve the quality of medical education at distributed campuses.

## Figures and Tables

**Table 1:** Qualitative data analysis codes grouped by theme, generated from *Dedoose* software

**Academic centre vs. distributed sites**			
academic vs. distributed sites	solo practice		
**Interest in teaching**			
perspectives on being a preceptor	past teaching experiences	family vs specialist teaching	interest in teaching
**Financial considerations**			
financial considerations			
**Administrative barriers**			
hospital administration barriers	work environment and space	medical school administration barriers	
**Medical experience and knowledge currency**			
teaching about ambiguous cases	medical knowledge	their own medical education	
**Practice environment and schedule**			
excuses not to teach	practice is too busy	rhythm of practice/time since school	patient perspective
takes time away from family obligations	teaching takes time/slows you down	scheduling	
**Training on teaching**			
availability of CME	teaching about teaching	challenges as a new preceptor	experience teaching
teaching experience in residency			
**Setting up systems for learners in distributed campus settings**			
rewards of teaching	unsure of learner roles	suggestions to improve teaching	setting up systems for learners
**Student engagement and medical learner level**			
learners that are interested	teaching medical students versus residents	challenges as a learner	

## Appendix A

Guiding questions and prompts for semi-structured open-ended interview.

Q1: To begin, I was just hoping to get an idea of your scope of medical practice. Could you spend a few moments describing your practice and what your day to day medical work involves? (Probe: practice setting - individual, team, or hospital-based; have you always had this scope of practice or has it changed? What aspects are the most challenging/enjoyable?)

Q2: I was just hoping to get an idea whether you are currently involved in any teaching, this could mean clinical, of medical students and residents, or in an academic sense, including lecturing, facilitating tutorial or Pro-Comp etc.? If you are not currently involved, have you been involved in any in the past? (Probe: in residency/after residency, non-medical teaching)

Q3: How do you personally feel about teaching? (Probe: have you been involved in any teaching? Did you find it: enjoyable? Challenging? Boring? Draining? Do you feel that it is important to teach?; how have your own experiences being taught shaped your perspectives on teaching? Good experiences as student? Bad experiences as student?)

Q4: What are some reasons that you would consider teaching? (Probe: If you have thought about it, why have you not pursued this further? If you have not even though about it, why is that?)

Q5: What are some barriers that prevent you from teaching? (Probe: if financial reasons is the primary barrier, given that teaching stipends are not stipulated by the provincial government and not alterable, what barriers would there be to teaching if money wasn’t the issue? personal or professional barriers? Administrative issues from the WRC that act as barriers?)

Q6: Are there any accommodations that might make teaching more enticing, or less of a burden for you? (Probe: by whom? Elaborate, if financial, how much? Are there any professional development sessions that could be offered to better accommodate you and other physicians to teach?)
